# Novel involvement of LMTK2 and EML6 in rheumatoid arthritis: potential biomarkers for disease activity and seronegative patients

**DOI:** 10.3389/fimmu.2026.1751440

**Published:** 2026-02-11

**Authors:** Hubert Kubis, Marek Cieśla, Anna Pałka, Bogdan Kolarz

**Affiliations:** 1 Laboratory of Diagnostic and Clinical Epigenetics, Faculty of Medicine, Collegium Medicum, University of Rzeszów, Rzeszów, Poland; 2 Department of Medical Biology, Faculty of Medicine, Collegium Medicum, University of Rzeszów, Rzeszów, Poland; 3 Department of Rheumatology, Faculty of Medicine, Collegium Medicum, University of Rzeszów, Rzeszów, Poland

**Keywords:** DAS28, EML6, LMTK2, rheumatoid arthritis, RNA-seq

## Abstract

**Introduction:**

Rheumatoid arthritis (RA) is a chronic, multifactorial autoimmune disease characterized by symmetrical polyarthritis, joint pain, and swelling, which can lead to disability and premature death. Increasing attention has focused on epigenetic mechanisms, including non-coding RNAs in circular and linear forms, in RA pathogenesis.

**Purpose:**

This study aimed to identify novel supportive RNA-based biomarkers associated with RA disease activity.

**Patients and methods:**

The discovery cohort included 18 RA patients and 10 healthy controls (HCs). Peripheral blood mononuclear cells were analyzed using targeted RNA sequencing, encompassing both linear (LINOUT) and circular (CIRC) RNA forms, to assess differences between RA patients and HCs, as well as between high (HDA; DAS28 > 5.1; n = 10) and low disease activity/remission (LDA/REM; DAS28 ≤ 3.2; n = 8) disease activity groups. The results were validated in cohort of 45 patients with RA, divided into a high disease activity group (HDA; DAS28 > 5.1; n = 22) and a non-high disease activity group (non-HDA; DAS28 ≤ 5.1; n = 23) along with 24 control subjects, using quantitative PCR (qPCR).

**Results:**

LMTK2 LINOUT expression correlated negatively with disease activity $(r_{s}\;$= -0.30) and distinguished RF-negative patients (n = 17) from HCs (p = 0.027). Expression was significantly lower in the high activity group (n = 22) versus the non-high activity group (n = 23; p = 0.022). *Post-hoc* ANOVA showed significant differences among HDA, non-HDA, and HCs (p = 0.001), with reduced expression in HDA versus non-HDA (p = 0.038) and increased expression in non-HDA versus controls (p = 0.001). EML6 LINOUT expression exhibited activity-dependent changes (p = 0.034) and positively correlated with disease activity $(r_{s}\;$= 0.303). Integration with erythrocyte sedimentation rate (ESR) improved discriminative performance. Combining LMTK2 LINOUT + EML6 LINOUT + ESR yielded the highest accuracy (AUC = 0.923).

**Conclusion:**

LMTK2 and EML6 show disease activity-dependent expression in RA and provide complementary information to conventional inflammatory parameters such as ESR. Their integration may improve diagnostic performance, highlighting their potential as novel supportive molecular biomarkers in RA.

## 1 Introduction

Rheumatoid arthritis (RA) is a multifactorial, chronic autoimmune disease characterized by symmetrical polyarthritis, joint pain, and swelling, which leads to disability and, if untreated, may result in premature mortality (1). A hallmark of the disease is chronic inflammation characterized by the involvement of immune system cells, particularly macrophages and T lymphocytes, in addition to resident joint cells (2, 3). As with other autoimmune diseases, the physiological immune response to autoantigens in RA leads to tissue damage, which, in the case of RA, includes the formation of pannus tissue (4). The disease affects both males and females across various age groups. RA most commonly manifests between 50 and 60 years of age but, may occur at any age, and it is 2–3 times more frequently in women (5, 6). It is estimated that by 2050, approximately 31.7 million individuals worldwide will be affected by this condition (7). The pathogenesis of RA is complex and involves genetic, epigenetic, and environmental factors that contribute to the development, persistence, and exacerbation of the disease (8, 9).

Standard assays detecting circulating rheumatoid factor (RF) and anti-cyclic citrullinated peptide antibodies (ACPA) represent the principal serological markers included in the 2010 ACR/European League Against Rheumatism (EULAR) classification criteria for RA diagnosis However, approximately one-third of RA patients are seronegative, which complicates effective diagnosis (10). Despite current diagnostic criteria, there remains a significant unmet need for novel, more sensitive biomarkers for RA, particularly for seronegative patients, representing a major ongoing challenge.

Non-coding RNAs (ncRNAs) have recently emerged as a central focus of research on gene expression regulation, playing critical roles in various biological processes, including immune regulation, inflammation, and cellular differentiation (11). NcRNAs constitute a diverse class of RNA transcripts that are not translated into proteins. However, recent studies challenge this traditional view, revealing that certain ncRNAs can be translated into functional peptides or microproteins (12, 13). Linear ncRNAs are classified into short and long RNA molecules. Among the short RNAs, microRNAs (miRNAs) are the most extensively studied group, whereas long non-coding RNAs (lncRNAs) represent the most thoroughly investigated category among the long ones. The latter can be further categorized based on their structural conformation into linear and circular forms, the latter commonly referred to as circular RNAs (circRNAs) (14). The biogenesis of circRNAs involves back-splicing via three distinct mechanisms: pairing-driven circularization, lariat-driven circularization, and RNA-binding protein (RBP)-driven circularization. circRNAs lack free 3’ and 5’ ends, instead forming covalently closed loop structures (15). Recent bioinformatic analyses have expanded our understanding of circRNAs’ cellular roles, which include protein interactions, transcriptional regulation, direct binding to mRNAs, and their potential to be translated. The most frequently described function of circRNAs is their role as miRNA sponges. CircRNAs are ubiquitously expressed in various tissues relevant to RA, such as cartilage, synovial tissue, and connective tissue, as well as in peripheral blood components including whole blood, serum, and peripheral blood mononuclear cells (PBMCs) (16). The prolonged stability of circRNAs in bodily fluids, alongside their diverse biological functions, positions them as a promising group of biomarkers in RA (15, 17).

Although thousands of circRNAs have been identified using RNA-seq data, their detection and characterization remain challenging. CircRNAs constitute only a small fraction of the total RNA pool and are typically expressed at low levels (18). Moreover, they share the same sequence as their linear counterparts, making them difficult to distinguish (19). Although numerous bioinformatic tools have been developed for circRNA identification and quantification, their performance and reproducibility across different approaches remain limited (18, 20, 21).

Current research focuses on the differential expression of linear and circular isoforms of the same ncRNA transcripts, as well as changes in their relative ratios in PBMCs of patients with RA.

## 2 Materials and methods

### 2.1 circRNA research panel

The Ion AmpliSeq™ Circular RNA Research Panel (Thermo Fisher Scientific, Waltham, MA, USA) encompasses 271 amplicons corresponding to genes involved in key biological pathways. An additional analytical parameter—the ratio of the circular to total transcript forms [circular/(circular + linear)] was calculated to assess the imbalance between circular and linear isoforms. Subsequently, genes were selected for differential analysis if their median expression ratio between diseased and healthy samples exhibited a difference of at least ± 1.5.

### 2.2 RNA-seq screening

The study cohort for RNA-seq screening comprised 28 individuals, including 18 patients diagnosed with RA, with a mean age of 60.11 ± 6.09 years, of whom 77.78% were female, and 10 healthy controls (HCs) aged 58.8 ± 3.99 years, with 70% female. The study cohort comprised RA diagnosis was established according to the 2010 ACR/EULAR classification criteria. The characteristics of the patients were derived from medical records and laboratory test results. These data are presented in **Supplementary Table 1**. Patients were divided into two groups according to disease activity based on DAS28-ESR: a high disease activity group (HDA; DAS28 > 5.1; n = 10) and a low disease activity/remission (LDA/REM; DAS28 ≤ 3.2; n = 8), which included patients with low disease activity (n = 4; 50%) and remission (n = 4; 50%). The characteristics of the groups are presented in **Supplementary Table 2**. Only patients with high disease activity and those with low activity or remission were included in the screening phase; patients with moderate disease activity were excluded. This approach was chosen to maximize the clinical contrast between groups and to reduce the heterogeneity inherent to intermediate disease activities.

The control group consisted of healthy volunteers and patients with osteoarthritis recruited from the outpatient clinic. Exclusion criteria included a confirmed history of autoimmune diseases other than the primary condition, significant comorbidities, including active infections, malignancy, advanced heart failure, or end-stage renal disease. Pregnancy was also an exclusion criterion. All patients and HCs were recruited at a single center (The Clinical Provincial Hospital in Rzeszów, Poland). All participants were of the same ethnicity.

For validation of the results using qPCR, a cohort of 69 individuals, including 45 patients diagnosed with RA was utilized. The characteristics of this group are presented in **Table 1**. The inclusion and exclusion criteria were identical to those applied for the selection of individuals for RNA-seq. Patients were stratified into two groups: HDA as previously described (DAS28 > 5.1, n=22), and non-high disease activity (non-HDA, DAS28 ≤ 5.1, n=23), which encompassed patients with moderate (n = 7; 30.43%) and low disease activity (n = 9; 39.13%) as well as remission (n = 7; 30.43%). Detailed demographic and clinical characteristics of the qPCR Verification Group presented in **Tables 1**, **2**. Serum samples were used to assess the presence of rheumatoid factor (RF; Rheumatoid Factor IgG ELISA kit, Demeditec Diagnostics, Kiel, Germany) and anti-citrullinated protein antibodies (ACPA; EIA CCP IgG, TestLine Clinical Diagnostics, Brno, Czech Republic). Antibody levels were determined by measuring absorbance values using enzyme-linked immunosorbent assay (ELISA) and a BioTek 800 TS microplate reader (Agilent, Santa Clara, CA, USA).

**Table 1:** Characterization of the qPCR verification group.

Characteristics	RA, n=45	HCs, n=24	RA vs HCs p-value
Age, years	57 [51-64]	56 [50.5-60]	0.21
Females, n (%)	36 (80)	20 (83.33)	0.989
Duration of the disease, years	12 [6-21.5]	n/a	n/a
RF positive, n (%)	28 (70)	1 (4.16)	<0.001
ACPA positive, n (%)	33 (73.33)	0	<0.001
ESR, mm/h	17 [8-36]	9 [6.5-11]	0.007
DAS28	4.91 [3.01-5.92]	n/a	n/a
CDAI	21.45 ± 13.7	n/a	n/a
SDAI	22.805 [9.95-34]	n/a	n/a
VAS PGA	50 [40-70]	n/a	n/a
VAS PhGA	50 [28-70]	n/a	n/a
Swollen joints, median (IQR)	6 [2-14]	n/a	n/a
Tender joints, median (IQR)	2 [0-6]	n/a	n/a

Data are presented as, mean, mean ± SD; median [lower - upper quartile] or number (%). ACPA, anticitrullinated protein antibodies; DAS28, disease activity score 28; ESR, erythrocyte sedimentation rate; HCs, healthy controls; n/a, not applicable; RA, patients with rheumatoid arthritis; RF, rheumatoid factor.

**Table 2:** Characterization of the qPCR verification group based on DAS28 score.

Characteristics	HDA, n=22	non-HDA, n=23	HDA vs non-HDA p-value	HCs, n=24
Age, years	58 [52-61]	67 [51-67]	0.394090	56 [50.5-60]
Females, n (%)	17 (77.27)	19 (82.6)	0.9406	20 (83.33)
Duration of the disease, years	10 [6-20]	12 [7-25]	0.258846	n/a
RF positive, n (%)	14 (63.63)	14 (60.87)	0.9075	1 (4.16)
ACPA positive, n (%)	16 (72.72)	17 (73.91)	0.8047	0
ESR, mm/h	32 [19-65]	8 [5-16]	<0.001	9 [6.5-11]
DAS28	5.93 [5.52-6.31]	3.01 [2.22-3.56]	<0.001	n/a
CDAI	32.5468 ± 8.971	10.835 ± 7.582133	<0.001	n/a
SDAI	35 [29.53-39.6]	10 [5.2-14.66]	<0.001	n/a
VAS PGA	70 [50-70]	40 [20-50]	<0.001	n/a
VAS PhGA	68 [54-70]	28 [20-50]	<0.001	n/a
Swollen joints, median (IQR)	6 [3-8]	0	<0.001	n/a
Tender joints, median (IQR)	12 [10-17]	2 [0-4]	<0.001	n/a

ACPA, anticitrullinated protein antibody; CDAI, Clinical Disease Activity Index; csDMARD, conventional synthetic disease-modifying antirheumatic drug; DAS28, disease activity score of 28 joints; ESR, erythrocyte sedimentation rate; HCs, healthy controls; HDA, high disease activity; n/a, not applicable; non-HDA;, non-high disease activity; RA, rheumatoid arthritis; RF, rheumatoid factor; SDAI, Simplified Disease Activity Index; VAS PhGA, Visual Analog Scale-Physician Global Assessment; VAS PGA, Visual Analog Scale-Patient Global Assessments.

The levels of RF and ACPA had been determined previously. The detailed methodology is available in another publication (22).

The study was conducted in accordance with the principles outlined in the Declaration of Helsinki and received approval from the Bioethics Committee of the University of Rzeszow (protocol number 9/11/2020).

### 2.3 Total RNA extraction

Whole blood was collected in EDTA-coated tubes and centrifuged at 3600 rpm for 10 minutes to separate and remove plasma. Two tubes of plasma-depleted blood were topped up to their original volume with phosphate-buffered saline (PBS; EURx, Gdansk, Poland). The blood was then diluted 1:1 with PBS and transferred into Falcon tubes containing Gradisol (Aqua-Med., Łódź). Density gradient centrifugation was performed at 2100 rpm for 20 minutes to isolate PBMCs. Following centrifugation, the PBMCs were transferred to a new Falcon tube with PBS, centrifuged again at 1200 rpm for 5 minutes, and subsequently resuspended in NucleoZOL reagent (Macherey-Nagel, Düren, Germany). The cells were transferred into Eppendorf tubes, suspended in NucleoZOL, and stored at −80°C for further processing. Total RNA was isolated from PBMCs using the NucleoZOL Set according to the manufacturer’s protocol. RNA concentration was measured using a Qubit™ 4.0 Fluorometer and the RNA High Sensitivity (HS) Assay Kit (Thermo Fisher Scientific, Waltham, MA, USA).

### 2.4 cDNA library preparation and sequencing

An RNA input of 25 ng was reverse transcribed into cDNA using the SuperScript™ VILO™ cDNA Synthesis Kit (Thermo Fisher Scientific, Waltham, MA, USA), according to the manufacturer’s instructions. The cDNA library was prepared using the Ion AmpliSeq™ Circular RNA Research Panel (Thermo Fisher Scientific), Library preparation was performed automatically on the Ion Chef™ System using the DL8 Library Preparation Kit (Thermo Fisher Scientific, USA). After preparation, the libraries were pooled and subjected to emulsion PCR using the Ion Chef System and the Ion 540™ Kit-Chef. Sequencing was carried out on the Ion S5™ System utilizing Ion Torrent™ semiconductor sequencing technology (Thermo Fisher Scientific, Waltham, MA, USA).

### 2.5 Data analysis and normalization

Sequencing data were analyzed using the Coverage Analysis plugin in the Torrent Suite software 5.12.3. To ensure comparability of transcript levels across samples, a two-step normalization procedure was applied. First, the number of reads assigned to each transcript was divided by the total number of reads generated for the corresponding sample. Second, these values were normalized relative to the expression level of the reference gene β-actin. This approach enabled independent quantification of both circular and linear RNA transcript forms. In addition, the ratio of circular to total transcript forms was calculated based on the normalized expression levels of the respective circular and linear targets (circular form/circular form + linear form).

### 2.6 Quantitative analysis of targets

For the verification of selected targets, RNA was isolated from PBMCs using the same method as previously described. A total of 300 ng of RNA was used for reverse transcription with the SuperScript™ VILO™ cDNA Synthesis Kit (Thermo Fisher Scientific, Waltham, MA, USA), following the manufacturer’s instructions. The resulting cDNA samples were diluted 1:2 prior to qPCR analysis. qPCR reactions were performed using the SG qPCR Master Mix kit (EURx, Gdańsk, Poland) on a QuantStudio 5 thermocycler (Thermo Fisher Scientific). Thermocycling conditions were applied according to the manufacturer’s protocol, with 45 amplification cycles conducted for all targets. The annealing/elongation step was performed at 52 °C for 30 seconds. Following amplification, a melting curve analysis was conducted to confirm the specificity of the PCR products. The same sequence primers as those used in the Ion AmpliSeq™ Circular RNA Research Panel (Thermo Fisher Scientific) were applied in the qPCR reactions. A calibrator sample was included to normalize inter-plate variability. Beta-2-microglobulin (B2M) served as the reference gene for PBMC-derived cDNA. Data analysis was conducted using the QuantStudio™ Design & Analysis Software v1.5.2 (Thermo Fisher Scientific). Gene expression levels were calculated using the delta-delta Ct (ΔΔCt) method and are presented as relative expression levels (RQ).

### 2.7 Statistical analysis

The Shapiro-Wilk test was employed to assess the normality of data distribution. Quantitative variables exhibiting a normal distribution were presented as mean ± standard deviation (SD), whereas those not conforming to a normal distribution were described using the median along with the interquartile range (IQR). Differences between two independent groups were analyzed using either the Student’s t-test or the Mann-Whitney U test, depending on the distribution of the data. To compare the three groups, the Kruskal-Wallis one-way analysis of variance was employed, followed by a *post hoc* multiple comparison analysis. For categorical variables expressed as percentages, comparisons were made using contingency tables with the chi-squared (χ²) test and Yates’ correction where appropriate. The correlation between the expression level and diagnostic parameters was assessed using Spearman’s correlation, which was denoted as $r_{s}$.P-values less than 0.05 were considered statistically significant. The obtained p-values were adjusted using the Benjamini-Hochberg procedure (False Discovery Rate, FDR = 5%), to control the false discovery rate (FDR), thereby yielding q-values. Analyses were performed using the STATISTICA software package, version 13.3 (TIBCO Software Inc., Palo Alto, California, USA). and Graphpad Prism, version 8.0 (GraphPad Software, San Diego, CA, USA).

## 3 Results

### 3.1 The diagnostic potential of biomarkers distinguishing HCs from patients with RA using RNA-seq

**Figure 1:**
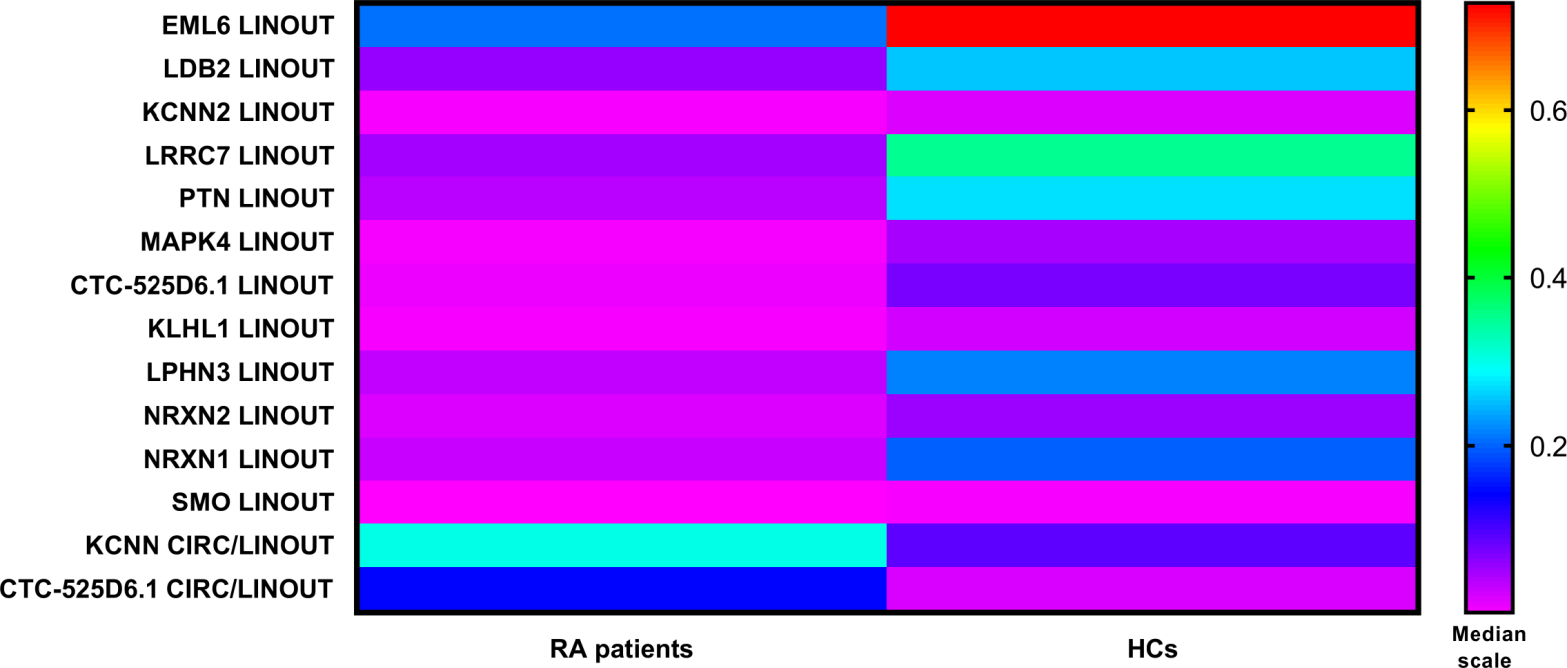
Relative targets expression based on the division into RA patients and HCs.

During the initial analysis of expression data, one patient was excluded due to low expression of the reference gene *β-actin*. Among the 271 genes analyzed, 26 exhibited 2.5-fold differential expression between patients with RA and HCs. After correction for multiple comparisons using the Benjamini-Hochberg method (q-value), the number of significantly dysregulated genes was reduced to 14. Mean total reads counts for these targets are presented in **Supplementary Table 4**. Of the selected targets, 12 were linear transcripts and 2 were defined based on the ratio of circular to total transcript forms. To facilitate visualization of expression differences, the median expression ratio between the RA and HCs group was calculated for each target. All 12 linear targets exhibited decreased expression, falling below the 0.5 threshold. In contrast, the median ratios of the two circular-to-linear targets were elevated in RA patients compared to HCs. The results are presented in **Table 3** and illustrated graphically in **Figure 1**.

**Table 3:** Differences in the expression of selected ncRNAs based on the division into RA patients and a HCs.

Target name	RA, n=17	HCs, n=10	p-value RA vs HCs	RA vs HCs q-value	Median ratio RA vs HCs
EML6 LINOUT	0.211 [0.187-0.316]	0.727 [0.604-0.968]	**<0.001**	**0.015**	0.29
LDB2 LINOUT	0.06 [0.048-0.102]	0.259 [0.193-0.432]	**<0.001**	**0.015**	0.23
KCNN2 LINOUT	0.004 [0.003-0.007]	0.02 [0.016-0.036]	**<0.001**	**0.007**	0.209
LRRC7 LINOUT	0.053 [0.036-0.076]	0.354 [0.227-0.767]	**0.002**	**0.013**	0.148
PTN LINOUT	0.039 [0.025-0.062]	0.274 [0.072-0.678]	**0.002**	**0.013**	0.143
MAPK4 LINOUT	0.006 [0.004-0.009]	0.05 [0.01-0.11]	**0.002**	**0.01**	0.114
CTC-525D6.1 LINOUT	0.01 [0.008-0.018]	0.076 [0.023-0.265]	**0.005**	**0.019**	0.132
KLHL1 LINOUT	0.005 [0.003-0.006]	0.026 [0.006-0.059	**0.005**	**0.019**	0.197
LPHN3 LINOUT	0.035 [0.021-0.046]	0.22 [0.086-0.868]	**0.006**	**0.017**	0.161
NRXN2 LINOUT	0.021 [0.015-0.039]	0.057 [0.034-0.149]	**0.006**	**0.017**	0.368
NRXN1 LINOUT	0.031 [0.019-0.038]	0.2 [0.039-0.584]	**0.011**	**0.026**	0.156
SMO LINOUT	0.0007 [0.0004-0.001]	0.005 [0.003-0.01]	**0.011**	**0.026**	0.16
KCNN CIRC/LINOUT	0.305 [0.26-0.536]	0.093 [0.012-0.255]	**0.017**	**0.033**	3.285
CTC-525D6.1 CIRC/LINOUT	0.148 [0.089-0.311]	0.021 [0.019-0.11]	**0.02**	**0.035**	6.915

The data presented refer to selected targets exhibiting at least a 2.5 -fold change in expression, either upregulated or downregulated, with selection based on statistical significance (p-value< 0.05). Data are presented as medians with interquartile ranges. Statistically significant values have been highlighted in bold. CIRC, circular form of transcripts; CIRC/LINOUT, the ratio of the circular to total transcript forms; HCs, healthy controls; LINOUT, linear form of transcripts; RA, rheumatoid arthritis.

Patients were stratified into RF-positive and RF-negative cohorts. The characteristics of the groups are presented in **Supplementary Table 3**. A threshold of a 2.5-fold change in gene expression was applied to define differentially expressed target genes. This criterion identified 100 candidate targets; however, only one gene, echinoderm microtubule-associated protein-like 6 (EML6 LINOUT), retained statistical significance following multiple testing correction using the Benjamini-Hochberg procedure (q-value). Detailed results are presented in **Table 4**.

**Table 4:** Differences in the expression of selected ncRNAs based on the division into presence RF.

Target name	RF positive, n=12	RF negative, n=5	HCs, n=10	p-value RF negative vs HC	p-value comparison of the three groups	q-value comparison of the three groups	*Post-hoc* ANOVA
EML6 LINOUT	0.286 [0.199 - 0.35]	0.156 [0.148 - 0.188]	0.727 [0.604 - 0.968]	0.003	**<0.001**	**0.04**	**RF positive vs HCs p=0.033** **RF negative vs HCs p<0.001**

The data presented refer to selected targets exhibiting at least a 2.5 -fold change in expression, either upregulated or downregulated, with selection based on statistical significance (p-value< 0.05). Data are presented as medians with interquartile ranges. Statistically significant values have been highlighted in bold. HCs, healthy controls; LINOUT, linear form of transcripts; RF, rheumatoid factor.

### 3.2 The diagnostic potential of biomarkers in distinguishing patients with HDA and non-HDA using RNA-seq

To ensure the selection of robust biomarkers with sufficiently high expression levels to enable reliable quantification and monitoring difference of expression, genes with<500 total reads were excluded from further analysis. The expression analysis revealed significant alterations in the expression of 21 genes in patients stratified according to the DAS28 score. Subsequent statistical evaluation using the Mann-Whitney U test identified lemur tyrosine kinase 2 (LMTK2 LINOUT) and LMTK2 CIRC/LINOUT transcripts as the only targets exhibiting a statistically significant difference in expression between patients with differing levels of disease activity. Detailed results are presented in **Table 5**. The total number of reads is available in **Supplementary Table 4**.

**Table 5:** Differences in the expression of selected targets based on DAS28 score.

Target name Target form	HDA, n=10	LDA/REM, n=7	HCs, n=10	p-value HDA vs non-HDA	p-value comparison of the three groups	*Post-hoc* ANOVA
APLF CIRC/LINOUT	0.928 [0.922-0.948]	0.933 [0.924-0.948]	0.863 [0.719-0.906]	0.733	**0.007**	**HDA vs HCs** **p=0.021** **LDA/REM vs HCs** **p=0.02**
APLF LINOUT	0.312 [0.237-0.401]	0.286 [0.187-0.336]	0.643 [0.49-0.959]	0.306	**0.004**	**HDA vs HCs** **p=0.046** **LDA/REM vs HCs** **p=0.005**
CTC-525D6.1 LINOUT	0.011 [0.008-0.022]	0.01[0.007-0.018]	0.076 [0.023-0.265]	0.526	**0.015**	**LDA/REM vs HCs** **p=0.02**
EML6 LINOUT	0.21 [0.156-0.316]	0.212 [0.188-0.323]	0.727[0.604-0.968]	0.661	**0.003**	**HDA vs HCs** **p=0.005** **LDA/REM vs HCs** **p=0.018**
HECTD1 CIRC/LINOUT	0.032 [0.028-0.039]	0.033 [0.024-0.039]	0.021 [0.021-0.024]	0.807	**0.014**	**HDA vs HCs** **p=0.024**
HOMER1 LINOUT	0.471 [0.376-0.595]	0.363 [0.339-0.384]	0.585 [0.499-0.790]	0.071	**0.018**	**LDA/REM vs HCs** **p=0.013**
IFT46 CIRC/LINOUT	0.646 [0.573-0.686]	0.61 [0.542-0.634]	0.453 [0.357-0.561]	0.306	**0.015**	**HDA vs HCs** **p=0.015**
IFT46 LINOUT	0.161 [0.105-0.181]	0.148[0.106-0.171]	0.295 [0.194-0.325]	0.591	**0.009**	**HDA vs HCs** **p=0.046** **LDA/REM vs HCs** **p=0.017**
KITLG LINOUT	0.166 [0.099-0.23]	0.221 [0.117-0.262]	0.46 [0.293-0.828]	0.591	**0.032**	**HDA vs HCs** **p=0.046**
LDB2 LINOUT	0.069 [0.048-0.105]	0.051 [0.048-0.102]	0.259 [0.193-0.432]	0.961	**0.002**	**HDA vs HCs** **p=0.009** **LDA/REM vs HCs** **p=0.009**
LMTK2 LINOUT	3.382 [2.496-4.837]	2.325 [1.875-2.906]	2.338 [1.52-3.268]	**0.045**	0.069	**n/a**
LMTK2 CIRC/LINOUT	0.104 [0.096 - 0.115]	0.144 [0.12-0.174]	0.138 [0.13-0.2]	**0.017**	0.07	**n/a**
LPHN3 LINOUT	0.042 [0.024-0.049]	0.031 [0.021-0.043]	0.22 [0.086-0.867]	0.407	**0.014**	**LDA/REM vs HCs** **p=0.016**
LRRC7 LINOUT	0.049 [0.03-0.102]	0.063 [0.044-0.076]	0.354 [0.227-0.767]	0.526	**0.008**	**HDA vs HCs** **p=0.013** **LDA/REM vs HCs** **p=0.046**
NRXN1 LINOUT	0.035 [0.022-0.05]	0.023 [0.019-0.035]	0.2 [0.039-0.585]	0.306	**0.02**	**LDA/REM vs HCs** **p=0.019**
PTN LINOUT	0.04 [0.026-0.088]	0.033 [0.023-0.0496]	0.274 [0.0717-0.678]	0.526	**0.006**	**HDA vs HCs** **p=0.05** **LDA/REM vs HCs** **p=0.008**
QSER1 CIRC/LINOUT	0.446 [0.420-0.496]	0.446 [0.395-0.48]	0.357 [0.288-0.439]	0.526	**0.025**	**HDA vs HCs** **p=0.026**
SCMH1 CIRC/LINOUT	0.961 [0.951-0.972]	0.949 [0.94-0.968]	0.927 [0.806-0.944]	0.354	**0.009**	**HDA vs HCs** **p=0.008**
SEPT10 CIRC/LINOUT	0.194 [0.117-0.264]	0.164 [0.142-0.208]	0.081 [0.017-0.177]	0.591	**0.03**	**HDA vs HCs** **p=0.034**
SEPT10 LINOUT	0.336 [0.242-0.504]	0.418 [0.316-0.428]	0.727 [0.519-1.174]	0.884	**0.014**	**HDA vs HCs** **p=0.036** **LDA/REM vs HCs** **p=0.041**
XPO1 LINOUT	20.708 [14.835-37.66]	0.376 [0.219-0.598]	0.853 [0.558-1.054]	0.526	**0.029**	**LDA/REM vs HCs** **p=0.041**
YY1AP1 LINOUT	0.694 [0.538-1.265]	0.506 [0.366-0.773]	1.152 [0.703-1.171]	0.188	**0.047**	**LDA/REM vs HCs** **p=0.041**
ZKSCAN1 LINOUT	0.143 [0.12-0.304]	0.147 [0.106-0.1617]	0.291 [0.187-0.488]	0.526	**0.035**	**LDA/REM vs HCs** **p=0.037**

The presented data were selected from samples with more than 500 total reads, with selection based on statistical significance (p-value< 0.05). Data are presented as medians with interquartile ranges. Statistically significant values have been highlighted in bold. CIRC, circular form of transcripts, CIRC/LINOUT, the ratio of the circular to total transcript forms HCs, healthy controls; HDA, high disease activity; LDA/REM, low disease activity/remission; LINOUT, linear form of transcripts; n/a, not applicable.

### 3.3 Verification of selected targets in an expanded study cohort using qPCR

Based on previous calculations, the targets EML6 LINOUT, LMTK2 LINOUT, and LMTK2 CIRC/LINOUT were selected for further analysis. To obtain the expression results for LMTK2 CIRC/LINOUT, the LMTK2 LINOUT and LMTK2 CIRC forms were analyzed, and then the ratio (RQ of the cyclic form/(RQ of the cyclic form + RQ of the linear form)) was calculated.

Among all the analyzed targets, only LMTK2 LINOUT exhibited statistically significant differences in expression between patients with RA and the control group. In the present study, a 1.3-fold increase in LMTK2 LINOUT expression was observed in RA patients compared to the control group. Detailed results are presented in **Table 6**. The characteristics of this groups are presented in **Table 1**.

**Table 6:** Differences in the expression of selected ncRNAs based on the division into on disease presence.

Target name Target form	RA, n=45	HCs, n=24	RA vs HCs p-value
EML6 LINOUT	0.885 [0.498-1.898]	1.14 [0.721-1.905]	0.456
LMTK2 LINOUT	0.98 [0.73 - 1.31]	0.758 [0.624-0.872]	**0.008**
LMTK2 CIRC	0.808 [0.639-1.091]	0.655 [0.452-1.029]	0.086
LMTK2 CIRC/LINOUT	0.476 [0.403-0.536]	0.444 [0.406-0.559]	0.955

Data are presented as medians with interquartile ranges. Statistically significant values have been highlighted in bold. CIRC, circular form of transcripts, CIRC/LINOUT, the ratio of the circular to total transcript forms HCs, healthy controls; LINOUT, linear form of transcripts; RA, rheumatoid arthritis.

Subsequently, patients with RA were divided into groups based on disease activity. Expression level of LMTK2 LINOUT was more than 1.5-fold higher in individuals with non-HDA compared to those with HDA, and more than 1.6-fold higher compared to the control group (p = 0.001). Details are presented in **Table 7**.

**Table 7:** Differences in the expression of selected ncRNAs based on the division into DAS28 score.

Target name	HDA, n=22	non-HDA, n=23	HCs, n=24	p-value HDA vs non-HDA	p-value comparison of the three groups	*Post-hoc* ANOVA
EML6 LINOUT	1.128 [0.68-2.757]	0.699 [0.392-1.458]	1.14 [0.721-1.905]	**0.034**	0.08	n/a
LMTK2 LINOUT	0.7885 [0.605-1.236]	1.221 [0.863-1.451]	0.758 [0.624-0.872]	**0.022**	**0.001**	**HDA vs non-HDA** **p=0.038** **non-HDA vs HCs** **p=0.001**
LMTK2 CIRC	0.777 [0.589-1.037]	0.867 [0.656-1.272]	0.655 [0.451-1.029]	0.212	0.1	n/a
LMTK2 CIRC/LINOUT	0.474 [0.442-0.548]	0.476 [0.383-0.536]	0.444 [0.406-0.559]	0.503	0.728	n/a

Data are presented as medians with interquartile ranges. Statistically significant values have been highlighted in bold. CIRC, circular form of transcripts, CIRC/LINOUT, the ratio of the circular to total transcript forms HCs, healthy controls; HDA, high disease activity; LINOUT, linear form of transcripts; n/a, not applicable; non-HDA, non-high disease activity.

On the other hand, stratification of patients based on RF status revealed significant differences in LMTK2 LINOUT expression between RF-negative individuals and the control group. RF-negative participants exhibited a 1.33-fold higher (p=0.027) expression level of LMTK2 LINOUT compared to controls. Additionally, LMTK2 expression differed significantly among RF-positive patients, RF-negative patients, and the control group. Detailed information is presented in **Table 8**.

**Table 8:** Differences in the expression of selected ncRNAs based on the division into RF negative patients and HCs.

Target name	RF positive, n=28	RF negative, n=17	HCs, n=24	p-value RF negative vs HCs	p-value comparison of the three groups	Post -hoc ANOVA
EML6 LINOUT	0.997 [0.593-1.971]	0.809 [0.392-1.66]	1.14 [0.721-1.905]	0.345	0.389	n/a
**LMTK2 LINOUT**	0.961 [0.675-1.335]	1.01 [0.734-1.31]	0.758 [0.624-0.872]	**0.027**	0.101	n/a
LMTK2 CIRC	0.914 [0.62-1.234]	0.741 [0.639-0.981]	0.785 [0.624-0.872]	0.46	**0.02**	**RF negative vs HCs** **p=0.015**
LMTK2 CIRC/LINOUT	0.486 [0.416-0.555]	0.455 [0.347-0.522]	0.444 [0.406-0.559]	0.396	0.21	n/a

Data are presented as medians with interquartile ranges. Statistically significant values have been highlighted in bold. HCs, healthy controls; LINOUT, linear form of transcripts; n/a, not applicable; RF, rheumatoid factor.

Subsequently, the correlation between the analyzed targets and diagnostic parameters used in the assessment of RA was investigated. The molecular marker LMTK2 LINOUT exhibited a negative correlation with several clinical parameters, including tender joint count ( $r_{s}\;$= -0.37), Visual Analog Scale-Physician Global Assessment (VAS PhGA) ( $r_{s}$= -0.40), DAS28 score ( $r_{s}$= -0.30), Simplified Disease Activity Index (SDAI score) ( $r_{s}$ = -0.40), and CDAI score ( $r_{s}$ = -0.33). LMTK2 CIRC exhibited a negative correlation with swollen joint count ( $r_{s}$= -0.35) and Visual Analog Scale-Patient Global Assessments (VAS PGA) ( $r_{s}$ = -0.30), whereas LMTK2 CIRC/LINOUT showed a positive correlation with VAS PGA ( $r_{s}$ = 0.30). Detailed information is presented in **Table 9**.

**Table 9:** The correlation between diagnostic parameters and the expression of selected target.

Target name	ESR	Tender joints	Swollen joints	VAS PGA	VAS PhGA	DAS 28	SDAI	CDAI	RF	ACPA
EML6 LINOUT	0.27	0.201	0.056	0.077	0.242	**0.303**	0.266	0.222	0.135	0.166
LMTK2 LINOUT	-0.078	**-0.37**	-0.215	-0.157	**-0.4**	**-0.308**	**-0.399**	**-0.327**	-0.087	0.054
LMTK2 CIRC	-0.077	-0.221	**-0.351**	**-0.298**	-0.197	-0.151	-0.229	-0.2	0.084	0.181
LMTK2 CIRC/LINOUT	-0.019	0.062	**0.305**	0.172	0.121	0.095	0.093	0.022	0.162	0.143

ACPA, anticitrullinated protein antibody; CDAI, Clinical Disease Activity Index; DAS28, disease activity score of 28 joints; ESR, erythrocyte sedimentation rate; n/a, not applicable; RA, rheumatoid arthritis; RF, rheumatoid factor; SDAI, Simplified Disease Activity Index; VAS PhGA, Visual Analog Scale-Physician Global Assessment; VAS PGA, Visual Analog Scale-Patient Global Assessments.Statistically significant correlations are highlighted in bold.

The logistic regression was used to measure the Area Under the Curve (AUC) for the analyzed targets, clinical parameters, and combinations of the analyzed targets with clinical parameters. Detailed information is presented in **Table 10**. Receiver operating characteristic (ROC) curves, along with the optimal cut-off values for LMTK2 LINOUT and EML6 LINOUT, in differentiating patients with HDA vs non-HDA activity and RF-negative patients from HCs, are provided in **Supplementary Figure 1**.

**Table 10:** Assessment of the predictive value of the studied markers.

Molecular markers	
RF negative (n=17) vs HCs (n=24)	
Target name	AUC
EML6 LINOUT	0.60
LMTK2 LINOUT	0.71
LMTK2 CIRC	0.59
LMTK2 CIRC/LINOUT	0.56
RF positive (n=28) vs RF negative (n=17)	
Target name	AUC
EML6 LINOUT	0.59
LMTK2 LINOUT	0.52
LMTK2 CIRC	0.61
LMTK2 CIRC/LINOUT	0.61
HDA (n=22) vs non-HDA (n=23)	
Target name	AUC
EML6 LINOUT	0.686
LMTK2 LINOUT	0.7
LMTK2 CIRC	0.61
LMTK2 CIRC/LINOUT	0.559
CLINICAL PARAMETERS	
HDA (n=22) vs non-HDA (n=23)	
Clinical Parameter Name	AUC
ESR	0.889
Swollen joints	0.925
Tender joints	0.939
VAS PGA	0.834
VAS PhGA	0.943
Clinical-molecular combination	
HDA (n=22) vs non-HDA (n=23)	
Name	AUC
ESR + EML6 LINOUT	0.903
ESR + LMTK2 LINOUT	0.913
ESR + LMTK2 CIRC	0.895
ESR + LMTK2 CIRC/LINOUT	0.889
ESR + EML6 LINOUT + LMTK2 LINOUT	0.923

ESR, erythrocyte sedimentation rate; HCs, healthy controls; HDA, high disease activity; non-HDA, non-high disease activity; RF, rheumatoid factor; VAS PhGA, Visual Analog Scale-Physician Global Assessment; VAS PGA, Visual Analog Scale-Patient Global Assessments.

## 4 Discussion

The research conducted within the framework of the present study provides, for the first time, evidence of the involvement of EML6 and LMTK2 in the pathogenesis of RA. Our findings indicate that the expression levels of LMTK2 LINOUT and EML6 LINOUT are associated with RA disease activity, and that alterations in their expression enable the distinction between HDA and non-HDA patients. Moreover, the expression level of LMTK2 LINOUT allows for differentiation between RF negative patients and HCs, indicating that it may hold diagnostic potential. LMTK2 is a Ser/Thr kinase and one of the transmembrane protein kinases anchored in the plasma membrane. LMTK2 is broadly expressed across human tissues (23). LMTK2 interacts with CDK5 and protein phosphatase 1 (PP1c), as well as with the cystic fibrosis transmembrane conductance regulator (CFTR) (24), myosin VI (25, 26) as well as with both anti- and pro-apoptotic proteins (27). These interactions indicate the diverse cellular functions of LMTK2, including roles in protein transcription, intracellular protein transport, cell differentiation, and apoptosis. Additionally, LMTK2 has been shown to participate in the regulation of inflammation by modulating pro-inflammatory factors and activating the Nrf2 signaling pathway in microglial cells. The study by Rui et al. demonstrated a significant decrease in the levels of inflammatory mediators iNOS, NO, COX-2, and PGE2, along with pro-inflammatory cytokines TNF-α, IL-1β, and IL-6, following exogenous induction of LMTK2 expression in LPS-stimulated BV2 microglial cells (28).

Previous study has demonstrated that knockdown of LMTK2 in CFBE41o- bronchial epithelial cells leads to alterations in the expression of thousands of genes, suggesting a broad spectrum of activity. Under unstimulated conditions, LMTK2 influences the expression of 2,506 genes, whereas following activation of the TGF-β1 pathway, this number rises to 4,162, indicating that LMTK2 functions both independently and in coordination with TGF-β1 signaling. Bioinformatic analyses revealed that LMTK2 is involved in pathways related to cell survival and death, development, and susceptibility to cancer (29). Altered LMTK2 expression has been reported, among others, in prostate cancer (30, 31). Such a wide spectrum of interactions suggests that LMTK2 may serve as a key integrative element of multiple regulatory pathways, influencing cellular homeostasis and pathological processes. LMTK2 regulates Wnt/β-catenin signaling via GSK-3β in hepatocellular carcinoma (32). Additionally, it functions as a regulator of the RUNX3/Notch signaling pathway in human glioblastoma (33).

EML6 is a molecule that has been comparatively less studied and is not well characterized. EML6 belongs to the EMAP (echinoderm microtubule-associated protein) family. Its biological functions remain incompletely understood. To date, it has been established that EML6 may be involved in mitotic spindle formation and in the regulation of microtubule dynamics (34). A substantial portion of the functions of all EMAP family members appears to be conserved due to the presence of common structural motifs, such as the HELP and WD40 domains, which are involved in various cellular processes. Interestingly, EML6 has been implicated in oncogenic processes in human cancers, primarily through mutations and gene fusions involving EML6 (35). In prostate cancer, EML6, together with RTN4 (Reticulon-4), has been identified as a target of miR-148a-3p and may contribute to tumor progression (36).

Current literature provides no evidence of EML6 or LMTK2 involvement in the pathogenesis of RA, underscoring the novelty of the present findings, however both the Wnt/β-catenin and Notch signaling pathways play important roles in RA. Wnt/β-catenin signaling is involved in pathological features such as cell maintenance, differentiation, proliferation, and self-renewal in RA (37). The involvement of the Wnt signaling pathway in RA may represent a potential mechanism for disease activation (38). In contrast, activation of Notch signaling stimulates cells such as synoviocytes and macrophages to secrete pro-inflammatory cytokines, thereby exacerbating disease in RA (39–42).

Expression analysis between RA patients and HCs revealed that among all analyzed targets, only LMTK2 LINOUT showed an increase in expression in RA patients, compared to the HCs. This suggests a potential association between LMTK2 LINOUT expression and the pathophysiology of RA.

Moreover, the expression levels of LMTK2 LINOUT varied significantly depending on disease activity in patients with RA. Patients with non-HDA exhibited higher levels of LMTK2 LINOUT compared to those with high disease activity, as well as when compared across the three study groups.

In RA, global disease activity is assessed from both the patient’s and the physician’s perspectives, most commonly using the visual analogue scale (VAS). Numerous studies have highlighted substantial discrepancies between subjective assessments of disease activity made by patients and those made by physicians. These differences stem from divergent evaluation criteria: patients tend to base their assessments on perceived pain intensity, fatigue, and emotional burden, whereas physicians place greater emphasis on objective indicators of inflammatory activity as well as clinical and laboratory findings (43–46). Additionally, the co-occurrence of other conditions, such as neuropathic pain (47), fibromyalgia (48), depression and anxiety (49) which contribute to the intensification of pain perception and whose symptoms are often misattributed to RA, may distort the assessment of global disease activity. This, in turn, can complicate the accurate interpretation of the patient’s condition and hinder optimal therapeutic decision-making (50).

Given the limitations of subjective assessments such as VAS PGA and PhGA, the application of molecular biomarkers like LMTK2 LINOUT may serve as a valuable complement to traditional clinical and laboratory indicators, including ESR. The integration of these molecular markers with classical parameters enhances the ability to discriminate between different levels of disease activity, as evidenced by an increase in the AUC value up to 0.923 in the combined assessment model. This value is comparable to classical clinical parameters such as the number of tender or swollen joints, but exceeds the patient’s subjective assessment. Nevertheless, the physician’s evaluation remains the gold standard in assessing disease activity. This study demonstrates the potential use of blood-derived biomarkers as a helpful tool for evaluating disease activity. These markers may be useful when a physician’s assessment of the patient’s health status is not possible.

In the RF-negative patients, the identification of novel biomarkers that could improve disease detection is imperative. Although RF-related differences in EML6 expression were observed in the RNA-seq screening, these findings were not confirmed in the qPCR validation and are therefore not interpreted as evidence of RF-dependent regulation. In this context, the LMTK2 demonstrates diagnostic potential. Data from the present study indicate that LMTK2 LINOUT levels are elevated in RF-negative patients compared to HCs. Diagnostic performance analysis using the AUC reveals that LMTK2 LINOUT achieves a moderate value of 0.71 in distinguishing RF-negative patients from HCs, supporting its potential utility as a biomarker in seronegative individuals. However, further studies are needed to confirm these observations. Since 2010, the ACR/EULAR classification criteria for RA have included, in addition to disease duration, joint involvement, and inflammatory markers, serological markers – particularly ACPA and RF levels (10). The sensitivity and specificity of RF in the diagnosis of RA vary depending on the disease stage, RF isotype, and detection method. The sensitivity of IgM-RF ranges from 41-66% in early RA and increases to 62-87% in advanced stages (51, 52). A meta-analysis including over 7,000 patients demonstrated that methods such as nephelometry and latex agglutination achieve the highest overall sensitivity (~69%) (51). Among isotype-specific assays, IgM-RF shows the greatest sensitivity (63%), whereas IgA-RF exhibits the highest specificity (91%) (53). The simultaneous presence of multiple RF isotypes further increases the likelihood of RA, enhancing the diagnostic value of combined isotype detection (54, 55). Although RF remains an important serological marker in RA diagnostics, its interpretation is constrained by limited specificity. Positivity for RF is observed in about 5-25% of individuals without clinical manifestations of rheumatoid arthritis, and its occurrence rises progressively with age (56).

The inclusion of innovative biomarkers, such as those exhibiting altered expression in both seronegative patients and HCs, including LMTK2, may enhance the effectiveness of contemporary diagnostics, particularly when traditional classification criteria based solely on serological markers are not always sufficient.

Although classical clinical parameters such as tender and swollen joint counts demonstrated higher standalone accuracy than the analyzed molecular markers, EML6 and LMTK2 are not intended to replace routine clinical assessment. Their potential value lies in their objective, blood-based nature, which is independent of examiner experience and patient-reported symptoms. Unlike joint counts, which may be influenced by comorbid pain syndromes, psychological factors, or inter-observer variability, molecular markers provide standardized and reproducible measurements. Importantly, the assessment of EML6 and LMTK2 expression can be simplified to targeted quantitative PCR assays, which are already widely available in clinical laboratories and suitable for routine use. Therefore, these markers may serve as complementary tools to conventional measures, particularly in complex clinical scenarios or when physical examination is limited. Thus, molecular markers should be considered as supportive tools for the clinical assessment of a patient’s condition.

The study has certain limitations. The RNA-seq analysis compared patients with markedly different levels of disease activity within a relatively small cohort, which may have influenced the results and partially explains the differences observed in the subsequent qPCR validation. Additionally, the small number of RF-negative patients in the RNA-seq cohort limits the strength of RF-stratified inferences at the discovery stage. Furthermore, the study population predominantly consisted of patients with established rheumatoid arthritis, with a median disease duration of 12 years, which may limit the generalizability of the findings to patients with early-stage disease. This research should be regarded as a basic exploratory study and may serve as a useful starting point for future investigations by other researchers. The study should also be extended to individuals in the pre−RA stage, as well as to other autoimmune conditions such as systemic lupus erythematosus or Sjögren’s syndrome.

## 5 Conclusions

The present study provides novel evidence supporting the involvement of LMTK2 LINOUT and EML6 LINOUT in the molecular landscape of RA. Integration of clinical and molecular parameters may enhance diagnostic precision and provide a more accurate reflection of disease activity, particularly in seronegative patients. The obtained results highlight the importance of a translational approach that combines multiple levels of biological information in the evaluation of rheumatoid arthritis. Further studies involving larger patient cohorts are necessary to confirm the clinical applicability of the analyzed biomarkers.

## Data Availability

The datasets presented in this study can be found in online repositories. The names of the repository/repositories and accession number(s) can be found below: https://repozytorium.ur.edu.pl/handle/item/11979, 11979.
