# Supplementary material for: Novel involvement of LMTK2 and EML6 in rheumatoid arthritis: potential biomarkers for disease activity and seronegative patients
Source: Front Immunol. 2026 Feb 11;17:1751440. doi: 10.3389/fimmu.2026.1751440 (PMC12932525; doi:10.3389/fimmu.2026.1751440)
Supplement: Supplementary file 1 [file Supplementaryfile1.docx]

Supplementary Material

# Supplementary Tables

**Supplementary Table 1.** Characterization of the study group selected for RNA-seq screening

| Characteristics | RA, n=18 | HCs, n=10 | RA vs HCs  p-value |
| --- | --- | --- | --- |
| Age, years | 60.11 ± 6.09 | 58,8 ± 3.99 | 0.19 |
| Females, n (%) | 14 (77.78) | 7 (70) | 1.00 |
| Duration of the disease, years | 14.81 ± 10.83 | n/a | n/a |
| RF positive, n (%) | 13 (72.22) | 2 (20) | 0.02 |
| ACPA positive, n (%) | 15 (83.33) | 0 (0) | <0.001 |
| ESR, mm/h | 22.00 [5-65] | 8.00 [6-16] | 0.13 |
| DAS28 | 4.33 ± 1.83 | n/a | n/a |
| CDAI | 15.00 [9.4-33.0] | n/a | n/a |
| SDAI | 21.84 ± 15.10 | n/a | n/a |
| VAS PGA | 45.42 ± 23.56 | n/a | n/a |
| VAS PhGA | 42.48 ±2 4.02 | n/a | n/a |
| Swollen joints, median (IQR) | 5.5 [ 2.00-11.00] | n/a | n/a |
| Tender joints, median (IQR) | 1.00 [0.00-5.00] | n/a | n/a |

*Data are presented as, mean, mean ± SD; median [lower – upper quartile] or number (%). Abbreviations: ACPA, anticitrullinated protein antibodies; DAS28, disease activity score 28; ESR, erythrocyte sedimentation rate; HCs, healthy controls; n/a, not applicable; RA, patients with rheumatoid arthritis; RF, rheumatoid factor*

**Supplementary Table 2.** Characterization of the study group selected for RNA-seq screening based on DAS28 scale

| Characteristics | HDA,  n=10 | LDA/REM,  n=8 * | p-value  HDA vs LDA/REM | HCs, n=10 |
| --- | --- | --- | --- | --- |
| Age, years | 60.4 ± 5.93 | 59.75 ± 6.67 | 0.81 | 58,8 ± 3.99 |
| Females, n (%) | 9 (90.00) | 5 (62.5) | 0.41 | 7 (70) |
| Duration of the disease, years | 11.65 ± 8.11 | 18.75 ± 12.98 | 0.15 | n/a |
| RF positive, n (%) | 7 (70.00) | 6 (75.00) | 0.77 | 2 (20) |
| ACPA positive, n (%) | 8 (80.00) | 7 (87.50) | 0.83 | 0 (0) |
| ESR, mm/h | 59.5 [26-69] | 5.00 [3.5-12.5] | 0.0002 | 8.00 [6-16] |
| DAS28 | 5.97 [5.38-6.15] | 2.30 [1.2-2.91] | 0.008 | n/a |
| CDAI | 29.5 [23-35] | 9.2 [5.00-9.95] | 0.04 | n/a |
| SDAI | 32.97 ± 10.25 | 7.93 ± 4.53 | 0.32 | n/a |
| VAS PGA | 56.3 ± 20.03 | 31.81 ±21.24 | 0.23 | n/a |
| VAS PhGA | 58.6 ± 16.55 | 22.56 ± 14.97 | 0.50 | n/a |
| Swollen joints, median (IQR) | 4.5 [1.00-6.00] | 0 | 0.004 | n/a |
| Tender joints, median (IQR) | 10.5 [6.00-16.00] | 2.00 [1.00-3.00] | <0.001 | n/a |

*Data are presented as, mean, mean ± SD; median [lower – upper quartile] or number (%). Abbreviations: ACPA, anticitrullinated protein antibodies; DAS28, disease activity score 28; ESR, erythrocyte sedimentation rate; HCs, healthy controls;* HDA, high disease activity; LDA/REM, low disease activity/remission;  *n/a, not applicable; RA, patients with rheumatoid arthritis; RF, rheumatoid factor.*

**Supplementary Table 3.** Characterization of the study group selected for RNA-seq screening based on the division into presence RF

| Characteristics | RF positive patients, n=13 | | RF negative patients, n=5 | p-value  RF positive vs RF negative | HCs, n=10 |
| --- | --- | --- | --- | --- | --- |
| Age, years | | 59.84 ± 6.52 | 60.8 ± 5.4 | 0.78 | 58,8 ± 3.99 |
| Females, n (%) | | 10 (76.92) | 4 (80.00) | 0.62 | 7 (70) |
| Duration of the disease, years | | 11.42 ± 8.46 | 23.6 ± 12.26 | 0.03 | n/a |
| ACPA positive, n (%) | | 13 (100.00) | 2 (40.00) | 0.02 | 0 (0) |
| ESR, mm/h | | 26.00 [11.00-65.00] | 18.00 [2.00-26.00] | 0.46 | 8.00 [6-16] |
| DAS28 | | 5.12 [2.81-5.55] | 5.94 [2.01-6.05] | 0.88 | n/a |
| CDAI | | 11.00 [9.0-26.00] | 23.00 [10.00-34.00] | 0.49 | n/a |
| SDAI | | 20.72 ± 15.60 | 24.76 ± 14.98 | 0.63 | n/a |
| VAS PGA | | 42.35 ± 24.71 | 53.4 ± 20.42 | 0.39 | n/a |
| VAS PhGA | | 39.73 ± 25.13 | 50.00 ± 21.51 | 0.43 | n/a |
| Swollen joints, median (IQR) | | 1.0 [0.00-4.00] | 2.0 [0.00-6.00] | n/a | n/a |
| Tender joints, median (IQR) | 5.0 [2.0-7.0] | | 11.0 [4.0-14.0] | n/a | n/a |

*Data are presented as, mean, mean ± SD; median [lower – upper quartile] or number (%). Abbreviations: ACPA, anticitrullinated protein antibodies; DAS28, disease activity score 28; ESR, erythrocyte sedimentation rate; HCs, healthy controls; n/a, not applicable; RA, patients with rheumatoid arthritis; RF, rheumatoid factor*

**Supplementary Table 4.** RNA-seq read data for selected targets

| Name of the target | Target occurrence form | Total target reads | Mapped reads |
| --- | --- | --- | --- |
| b-actin | NEGATIVE | 8769.037 | 8655210 |
| EML6 | LINOUT | 4773.222 |  |
| LDB2 | LINOUT | 1633.111 |  |
| KCNN2 | LINOUT | 159.8148 |  |
| LRRC7 | LINOUT | 2733.333 |  |
| PTN | LINOUT | 2166.074 |  |
| MAPK4 | LINOUT | 330.3704 |  |
| CTC-525D6.1 | LINOUT | 695.8519 |  |
| KLHL1 | LINOUT | 229.3333 |  |
| LPHN3 | LINOUT | 2464.963 |  |
| NRXN2 | LINOUT | 477.1852 |  |
| NRXN1 | LINOUT | 2048.481 |  |
| SMO | LINOUT | 234.9259 |  |
| KCNN | CIRC | 30.07407 |  |
|  | LINEOUT | 159.8148 |  |
|  | CIRC/LINEOUT | 0.188 |  |
| CTC-525D6.1 | CIRC | 22.62963 |  |
|  | LINEOUT | 695.8519 |  |
|  | CIRC/LINEOUT | 0.033 |  |
| LMTK2 | LINOUT | 21671.92593 |  |
| LMTK2 | CIRC | 3259.703704 |  |

*The data presented refer to selected targets. Total reads and mapped reads counts were presented as mean values. For further analysis, the data were normalized to the total number of reads per target and subsequently normalized using beta-actin as a reference gene. Statistically significant differences were reported for normalized data for circular and linear RNA forms, as well as for the ratio of normalized data circular to linear forms, calculated using the formula: normalized data circular form / normalized data circular form + normalized data linear form. Multiple hypothesis testing for circular, linear, and circular-to-linear ratios was corrected using the Benjamini-Hochberg procedure, and the resulting adjusted p-values were reported as q-values ( q-value<0.05).* *Abbreviations: CIRC, circular form of transcripts, CIRC/LINOUT, the ratio of the circular to total transcript forms; LINOUT, linear form of transcripts.*

**Supplementary Figure 1.** Receiver operating characteristic (ROC) curves illustrating the diagnostic performance of LMTK2 LINOUT and EML6 LINOUT in differentiating patients with high versus non-high disease activity (HDA vs non-HDA) (upper panels) and RF-negative patients from healthy controls (RF− vs HCs) (lower panels). The diagonal red line represents random classification. The optimal cut-off values were determined using the Youden index and are indicated by the green markers on each curve. Corresponding Youden index values and proposed cut-off points are shown within each panel.
