# Supplementary figures and images for: Novel involvement of LMTK2 and EML6 in rheumatoid arthritis: potential biomarkers for disease activity and seronegative patients
Source: Front Immunol. 2026 Feb 11;17:1751440. doi: 10.3389/fimmu.2026.1751440 (PMC12932525; doi:10.3389/fimmu.2026.1751440)

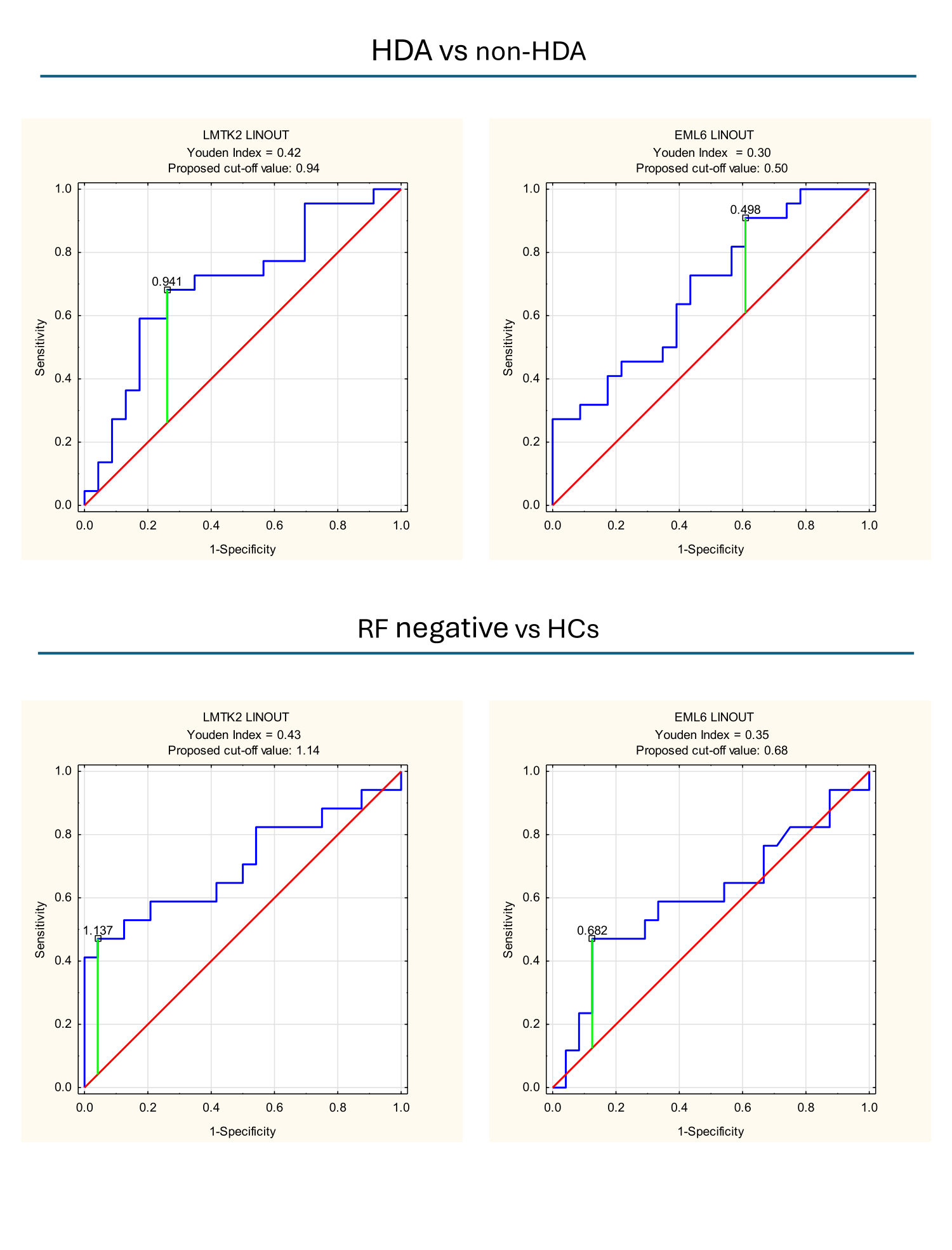

Supplement: Supplementary file 2 [file Image1.tiff]
